# African Trypanosomiasis Research at a Crossroads: Bibliometric and Systematic Insights for the Future

**DOI:** 10.1155/japr/9018223

**Published:** 2026-05-30

**Authors:** Israel Ogwuche Ogra, Jeremiah Zaphnathpaaneah Adaji, Emohchonne Utos Jonathan, Benita Kenechukwu Nwajiani, Samson Usman, Alexander Kurovsky, Kodjovi Sossou, Emmanuel Oluwadare Balogun

**Affiliations:** ^1^ Biological Institute, Tomsk State University, Tomsk, Russia, tsu.ru; ^2^ UNESCO International Centre for Biotechnology, Nsukka, Enugu State, Nigeria; ^3^ School of Health Sciences, Maduka University, Ekwegbe–Nsukka, Enugu State, Nigeria; ^4^ Department of Biochemistry, Federal University Wukari, Wukari, Taraba State, Nigeria, fuwukari.edu.ng; ^5^ Department of Zoology, Ahmadu Bello University, Zaria, Kaduna State, Nigeria, abu.edu.ng; ^6^ Department of Crop Protection, Ahmadu Bello University, Zaria, Kaduna State, Nigeria, abu.edu.ng; ^7^ LaSBASE: Laboratoire des Sciences Biomédicales, Alimentaires et de Santé Environnementale, Lome, Togo; ^8^ Department of Biochemistry, Ahmadu Bello University, Zaria, Kaduna State, Nigeria, abu.edu.ng

**Keywords:** bibliometrics, neglected tropical diseases, One Health, parasite, research output, VOSviewer

## Abstract

**Aim:**

Despite a reduction in the overall incidence and prevalence in the last two decades, trypanosomiasis continues to be a public health concern in Africa. This study was designed to comprehensively evaluate current trends in trypanosomiasis research using bibliometric approaches to uncover emerging topics and knowledge gaps, thereby guiding future investigations, particularly in Africa.

**Methods:**

Relevant articles were systematically selected from the Scopus‐retrieved dataset and analyzed using Microsoft Excel, VOSviewer, and the Bibliometrix software. A total of 4036 documents were analyzed.

**Results:**

The results revealed the involvement of 8849 authors, 696 sources, and an annual growth rate of 3.52%. A period of rapid increase in trypanosomiasis research was observed after 1973. Kenya and Nigeria were the leading countries in African trypanosomiasis research. The most relevant affiliation was Ahmadu Bello University, Nigeria, whereas the most relevant and impactful sources were the *Acta Tropica and PLOS Neglected Tropical Diseases*, respectively. Similarly, *Buscher, P*. emerged as the most relevant and impactful author in the field. Some notable keywords observed are *trypanocidal agents*, *prevalence*, *parasitemia*, and *polymerase chain reaction*. Furthermore, themes such as *antitrypanosomal* and *molecular docking* represented the recent research interests.

**Conclusion:**

This study provides a comprehensive overview of research output and evolution on trypanosomiasis in Africa and highlights critical ways forward in combating the disease, such as the One Health approach, strengthening health systems and capacities, and improving diagnostic and surveillance tools across African nations in addressing African trypanosomiasis. These findings are crucial for directing future research on African trypanosomiasis.

## 1. Introduction

Trypanosomiasis, commonly known as sleeping sickness in humans and *nagana* in animals, remains a significant public health and veterinary concern across Africa. The etiologies are protozoan parasites of the genus *Trypanosoma*. In humans, the disease is caused by two subspecies of *Trypanosoma brucei gambiense*, which is responsible for the chronic form prevalent in West and Central Africa, and *Trypanosoma brucei rhodesiense*, which causes the acute form in East and Southern Africa. In animals, particularly cattle, trypanosomiasis is caused by *Trypanosoma congolense, Trypanosoma vivax,* and *Trypanosoma brucei* and transmitted primarily by tsetse flies—*Glossina* species [1, 2]. The disease affects both human and animal populations, leading to substantial morbidity, mortality, and economic losses [3].

Although sustained control efforts have reduced the incidence by 97% in the last two decades, trypanosomiasis continues to pose a public health concern in Africa. According to reports, there is variation in the prevalence of trypanosomiasis across Africa, with significant differences observed between countries. In terms of human African trypanosomiasis (HAT) *T. b. gambiense* is responsible for over 92% of reported cases in 2023, predominantly affecting West and Central Africa, whereas *T. b. rhodesiense* accounts for about 8% of cases, mainly in East and Southern Africa. Reports further indicate that the Democratic Republic of the Congo (DRC) continues to bear the highest burden, reporting 61% of HAT cases over the last half‐decade, with an average of 522 cases annually. Some countries have reported fewer than 100 and 10 cases, respectively, whereas in others, only sporadic cases occur. Interestingly, no cases have been reported in a few countries for over a decade, suggesting possible interruption of transmission; however, the veracity of this needs to be fully ascertained [4].

In sub‐Saharan Africa, African animal trypanosomiasis (AAT) continues to pose a serious threat to livestock productivity and health. A meta‐analysis of research from 1960 to 2017 found that the overall prevalence of AAT in Nigeria was 16.1% across all livestock species and that trypanosome infection in tsetse flies, which are the disease′s main vectors, was 17.3% [5]. According to a systematic review and meta‐regression analysis conducted in Uganda between 1980 and 2022, the country′s AAT prevalence was 22.15% in cattle, 13.88% in goats, and 8.51% in sheep, with the northern and eastern regions having the highest prevalence [6]. These results highlight the enduring problem of trypanosomiasis in Africa and the necessity of ongoing study, vector management, and surveillance to lessen its effects on agricultural productivity, human health, and animal health.

Bibliometric analysis provides helpful information regarding research trends, productivity, and impact. It helps identify influential authors, key publications, and collaborative networks within a field. By analyzing citation patterns and publication data, researchers can uncover emerging topics and knowledge gaps, guiding future investigations [7]. Although bibliometrics, based on literature data, has been extensively employed across various fields to objectively and quantitatively assess interdisciplinary connections and unveil emerging topics [8], very few bibliometric studies are available on trypanosomiasis. Besides, these studies were conducted several years ago, which may not provide current insights into the status of research and current trends in the field of trypanosomiasis research. For instance, Thompson [9] analyzed 5139 articles on African trypanosomiasis published between 1900 and 1985, and Hassan et al. [10] examined global trypanosomiasis research from 1988 to 2017. The study highlighted that African countries, notably Nigeria and Kenya, were among the top contributors to trypanosomiasis research.

Due to the dearth of bibliometric research on trypanosomiasis in Africa, a comprehensive bibliometric analysis of trypanosomiasis research in Africa is necessary. It is important to analyze this research field in the African context since African trypanosomiasis is a disease affecting countries specifically in this continent. Such an analysis would provide valuable insights into research trends, identify underexplored areas, and inform future research priorities. By mapping the scientific landscape, stakeholders can better allocate resources, foster collaborations, and ultimately enhance the effectiveness of trypanosomiasis control and elimination efforts across the continent.

Therefore, the purpose of this study is to use bibliometric techniques to examine the research output on trypanosomiasis in Africa. To achieve this aim, we identified the general trends in trypanosomiasis research in Africa; evaluated country performance and global collaborations; analyzed the contributions of affiliations, sources, and authors; outlined the top most cited documents; showed the evolution of keywords; and discussed the way forward in the research field.

## 2. Methodology

### 2.1. Data Collection

**Figure 1:**
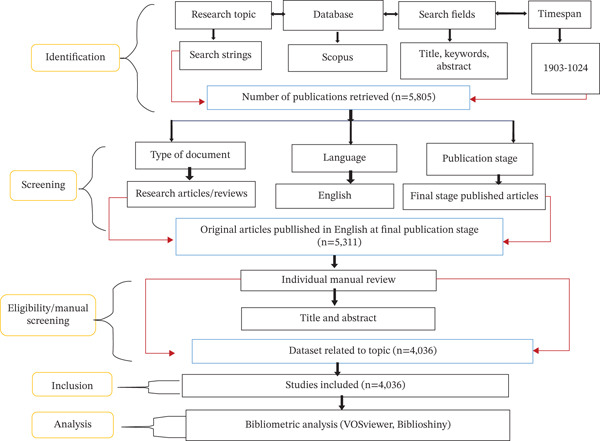
PRISMA model flow diagram.

The data sets used for this analysis were sourced and retrieved from the Scopus database on September 7, 2024 (Figure 1). This database is a comprehensive, multidisciplinary database indexing peer‐reviewed literature, offering tools for citation analysis and academic research tracking globally [11, 12]. The period was set at 1903–2024 to cover the onset of research output on the subject [13]. The search was performed on the titles, abstracts, and keyword fields using the search string (“Trypanosomiasis” OR “*Trypanosoma*” OR “sleeping sickness” OR “*Nagana*”) AND (“Africa”).

A total of 5805 results were retrieved. However, based on our inclusion criteria, we focused on research and review articles that were in English, resulting in a total of 5311 documents. The search results were exported as a CSV file for screening. The retrieved data were manually screened to systematically exclude publications that are not in line with the subject area. The Preferred Reporting Items for Systematic Reviews and Meta‐Analyses (PRISMA) guidelines were used for title and abstract screening and full‐text review, where necessary, following the exclusion criteria. A total of 4036 documents were selected and included as an exact depiction of relevant documents covering research on the search strings. This set of data was used for further analysis.

### 2.2. Data Analysis

The dataset retrieved from the Scopus search, which included 4036 publications after the manual screening, was stored in CSV format for subsequent analysis. The bibliometric analyses were conducted using the Bibliometrix and VOSviewer software, which allowed for the analysis of global trends in productivity, impact, and conceptual structure; the VOSviewer software generated network maps for keyword co‐occurrence and country coauthorship; and the data analysis was visualized using clustered bar charts in Microsoft Excel [14–16]. The bibliometric and systematic review approaches were integrated for the synthesis of insights, which involved the analysis of the most cited documents to generate key themes and concepts; all coauthors agreed to adhere to a common protocol (PRISMA) and apply consistent screening criteria; disagreements were resolved through discussion or consensus meetings.

## 3. Results

### 3.1. General Overview and Publication by Year

**Figure 2:**
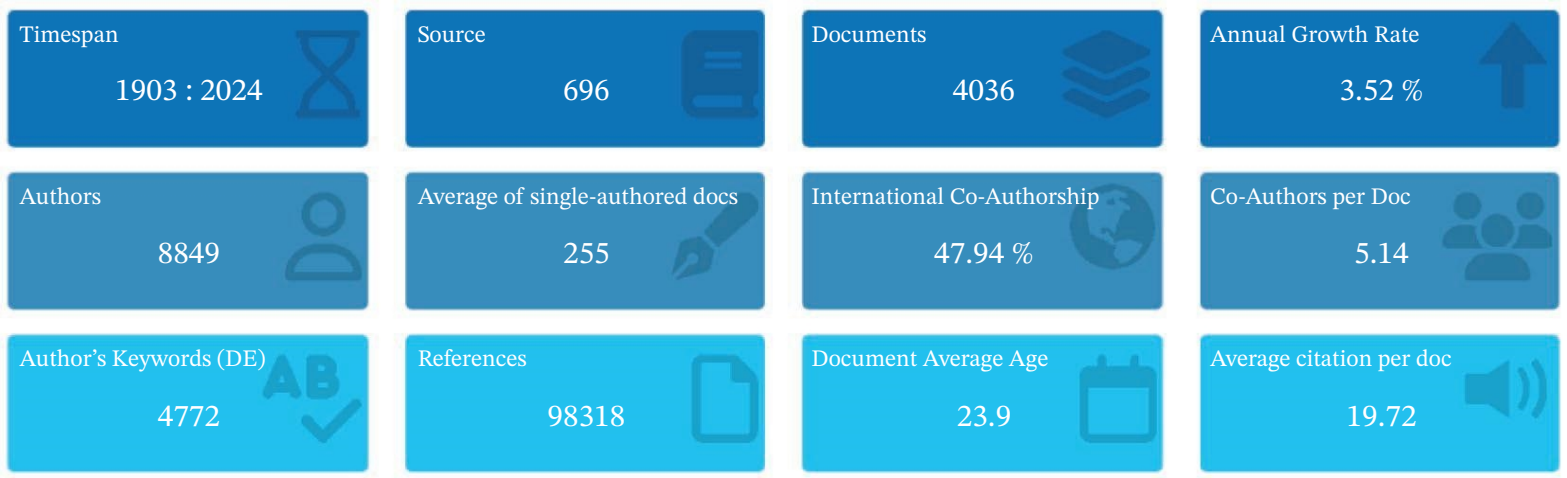
Main information on trypanosomiasis research in Africa.

The key details on the analyzed dataset are presented in Figure 2. The search for scientific information in the Scopus database revealed that a total of 4036 documents were published in 696 sources between 1903 and 2024. These publications were contributed by 8849 authors with diverse affiliations globally. With an annual rise of 3.52%, there is a considerable increase in the number of publications on research on trypanosomiasis in Africa within the given period.

**Figure 3:**
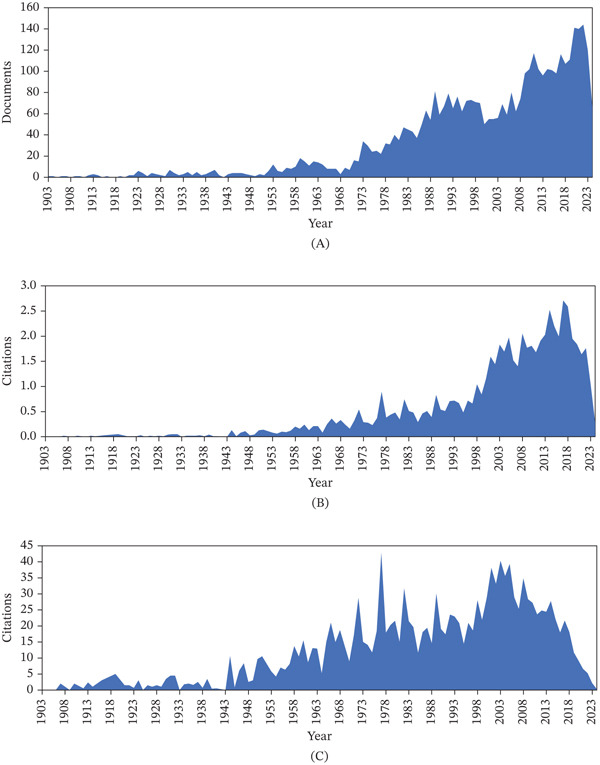
(A) Yearly distribution of scientific publications on trypanosomiasis research in Africa, (B) yearly average citations of scientific publications on trypanosomiasis research in Africa, and (C) average citations per article of scientific publications on trypanosomiasis research in Africa.

Figure 3A shows the yearly output of scientific publications on trypanosomiasis research in Africa from 1903 to 2024, with publications increasing steadily across the years despite fluctuations. Periods before 1973 had fewer than 20 research publications published annually. The obvious growth in publication was recorded in 1973, which continued to increase steadily despite slight yearly fluctuations observed. Figure 3B shows the yearly average citations of scientific publications on trypanosomiasis. The highest yearly average citation of scientific publications in the research field was observed in 2018, followed by 2013, with a slight decline after the year 2018 (Figure 3B). Similarly, the highest average total citations per article in the area were observed in 1973, which subsequently declined before peaking again at 2003 and further declining steadily, as shown in Figure 3C.

### 3.2. Country Performance and Collaborations

**Figure 4:**
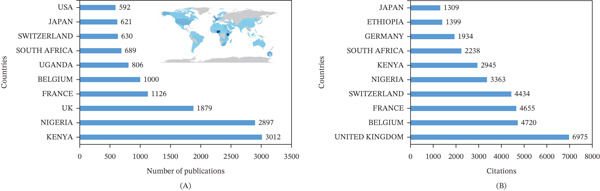
(A) The distribution of scientific publications on trypanosomiasis research in Africa and (B) the most cited countries of scientific publications on trypanosomiasis research in Africa.

The map in Figure 4A highlights the worldwide distribution of publications on trypanosomiasis. The deepest blue color on the world map represents countries with the highest number of publications, whereas the bar chart shows the Top 10 countries by scientific publications. Countries and regions in deep blue indicate areas with high research activity on the subject. According to the map, Kenya and Nigeria have the highest research output on trypanosomiasis. The distribution of documents across countries, as shown in Figure 4A, further corroborates that Kenya and Nigeria are the leading nations in research on trypanosomiasis in Africa. They are followed by three European nations of the United Kingdom, France, and Belgium. Two other countries in Africa, Uganda and South Africa, make the list of the Top 10 in terms of the highest number of publications, respectively. The last three are Switzerland, Japan, and the United States.

Figure 4B depicts the most cited countries of scientific publications in the field, with the United Kingdom, Belgium, France, Switzerland, and Nigeria ranking among the Top 5 performers in that category, respectively.

The most relevant countries of publications based on the corresponding author in terms of single‐country publications (SCP) and multiple‐country publications (MCP) are presented in Table 1. Nigeria ranks the highest with a total of 363 articles based on the corresponding author. Out of this, 82.6% are SCP, whereas 17.4% are MCP. The United Kingdom ranks second with 207 articles based on the corresponding author. However, out of this, 1.4% are SCP, and 98.6% are MCP. Others include Kenya, France, and Belgium, with 142, 136, and 134 articles, respectively, based on the corresponding author.

**Table 1:** Top 10 most relevant countries of publications based on the corresponding author in trypanosomiasis research in Africa and their collaboration nature.

Country	SCP	MCP	Articles	% SCP to total publications	% MCP to total publications
Nigeria	300	63	363	82.6	17.4
United Kingdom	3	204	207	1.4	98.6
Kenya	78	64	142	54.9	45.1
France	3	133	136	2.2	97.8
Belgium	1	133	134	0.7	99.3
South Africa	51	60	111	45.9	54.1
Ethiopia	64	32	96	66.7	33.3
Switzerland	2	86	88	2.3	97.7
Cameroon	13	67	80	16.2	83.8
Japan	0	71	71	0	100

### 3.3. Affiliations and Sources′ Contributions

**Figure 5:**
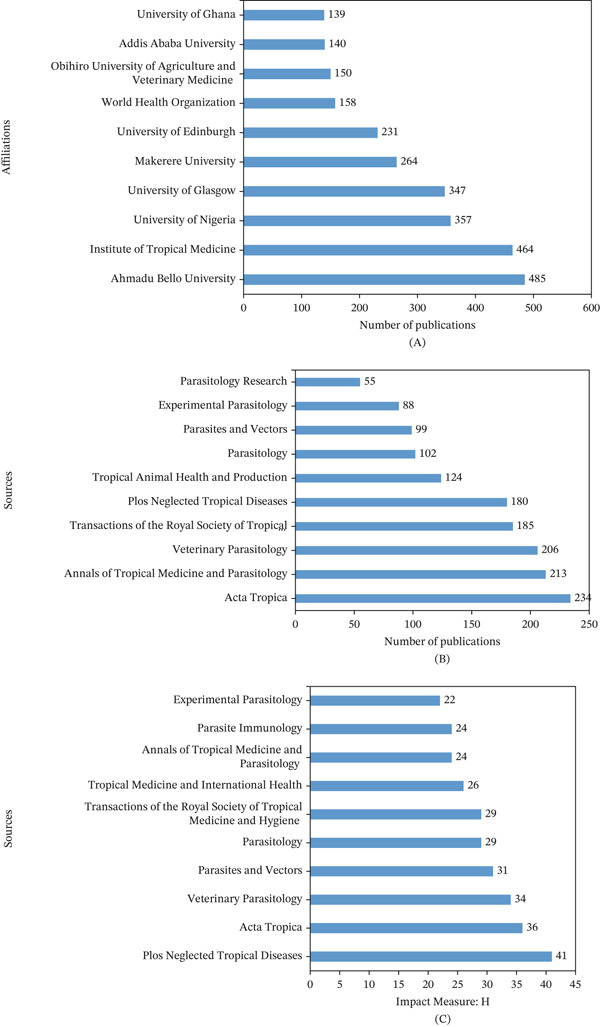
(A) The most relevant affiliations of scientific publications on trypanosomiasis research in Africa, (B) the Top 10 relevant sources of scientific publications on trypanosomiasis research in Africa, and (C) the most impactful sources of scientific publications on trypanosomiasis research in Africa.

Figure 5A shows the 10 leading affiliations in trypanosomiasis research in Africa. “Ahmadu Bello University” emerged as the most relevant affiliation, with a total of 485 documents. The next notable affiliation was “Institute of Tropical Medicine,” with 464 documents. These are followed by “University of Nigeria” (357 documents), “University of Glasgow” (347 documents), and “Makerere University” (264 documents). Others include “University of Edinburgh” (231), “World Health Organization” (158), and “Obihiro University of Agriculture and Veterinary Medicine” (150 documents). “Addis Ababa University” and “University of Ghana” make the last two of the Top 10 with 140 and 139 documents, respectively.

The Top 10 relevant sources of scientific publications out of the 696 sources (Figure 2) in research on trypanosomiasis, ranked according to the number of documents, are presented in Figure 5B. *Acta Tropica,* with a total of 234 documents, is the most relevant source for trypanosomiasis‐related studies in Africa. Other relevant sources include *Annals of Tropical Medicine and Parasitology* (213 documents), *Veterinary Parasitology* (206 documents), *Transaction of the Royal Society of Tropical Medicine and Hygiene* (185 documents), and *PLOS Neglected Tropical Diseases* (180 documents).

Figure 5C shows the Top 10 sources based on *h*‐index in research on trypanosomiasis. *PLOS Neglected Tropical Diseases* emerged as the leading source with an impact measure of 41, followed by *Acta Tropica* with an impact measure of 36. Others include *Veterinary Parasitology* (34*), Parasites and Vectors* (31), *and Parasitology* (29). *Parasite Immunology* and *Experimental Parasitology* were the last two among the Top 10 impactful sources of scientific publications on trypanosomiasis research in Africa with impact measures of 24 and 22, respectively.

### 3.4. Author′s Productivity and Impact

**Figure 6:**
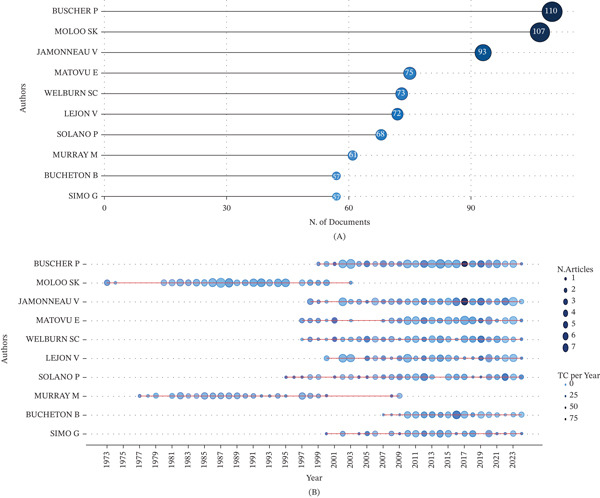
(A) The most relevant authors of scientific publications on trypanosomiasis research in Africa, and (B) top authors′ productivity over time from 1973 to 2024.

Furthermore, this study showed that 8849 authors contributed to publications in the research field, with 255 of them producing single‐authored documents (Figure 2). The Top 10 relevant authors according to the number of documents are presented in Figure 6A. Buscher, P. had the highest number of publications (110), followed by Moloo, S.K., with 107 publications. Jamonneau, V., Matovu, E., and Welburn, S.C., with 93, 75, and 73 publications, respectively, complete the list of the Top 5 authors with the highest scientific publications on trypanosomiasis research in Africa.

Figure 6B showcases the productivity of the Top 10 productive authors from 1973 to 2024 (representing the significant research growth phase in the field). Buscher, P.′s productivity spanned from 1999 to 2024, whereas Moloo, S.K.′s publication encompasses 1973 to 2003. Jamonneau, V*.,* had publications from 1998 to 2024. Both Matovu, E. and Welburn, S.C. had publications within the same time period of 1997–2024. The results show that 8 among the Top 10 relevant authors are still actively involved in trypanosomiasis research.

Table 2 presents the authors with the highest impact in the field. Results show that Buscher, P., sits atop with an *h*‐index of 35, whereas Jamonneau, V., follows closely with an index of 34. Lejon, V., Solano, P., and Welburn, S.C. follow with *h*‐indices of 30, 29, and 29, respectively.

**Table 2:** Top 10 authors with the highest impact in trypanosomiasis research in Africa.

Author	*h*‐index	*g*‐index	*m*‐index	TC	NP	PY_start
Büscher P.	35	59	1.346	4056	110	1999
Jamonneau V.	34	54	1.259	3179	93	1998
Lejon V.	30	47	1.2	2381	72	2000
Solano P.	29	44	0.967	2121	68	1995
Welburn S.C.	29	50	1.036	2722	73	1997
Cuny G.	27	40	0.931	1670	48	1996
Bucheton B.	26	41	1.444	1801	57	2007
Cecchi G.	24	43	1.5	2554	43	2009
Franco J.R.	24	33	0.923	2409	33	1999
Moloo S.K.	24	37	0.462	1985	107	1973

Abbreviations: NP, number of publications; PY_start, publication year start; TC, total citations.

### 3.5. Topmost Cited Documents

The most cited publications in the field are presented in Table 3. Hirumi and Hirumi [17], working on continuous cultivation of *T*. *brucei* bloodstream forms in a medium containing a low concentration of serum protein without feeder cell layers, published in the *Journal of Parasitology, which* was the most cited publication with 826 citations. This is followed by Murray et al. [18] with a total citation of 531. Others include Buscher et al. [19], Priotto et al. [20] and Masiga et al. [21], with 487, 420, and 333 citations, respectively, concluding the list of the Top 5 most cited documents on trypanosomiasis research within the given period.

**Table 3:** Description of the Top 10 most cited documents in trypanosomiasis research in Africa.

S/No	Author	Title	Journal	DOI	Total citations
1	Hirumi and Hirumi [17]	Continuous Cultivation of *Trypanosoma brucei* Blood Stream Forms in a Medium Containing a Low Concentration of Serum Protein Without Feeder Cell Layers	*Journal of Parasitology*	10.2307/3282883	826
2	Murray et al. [18]	An Improved Parasitological Technique for the Diagnosis of African Trypanosomiasis	*Transactions of the Royal Society of Tropical Medicine and Hygiene*	10.1016/0035‐9203(77)90110‐9	531
3	Büscher et al. [19]	Human African Trypanosomiasis	*The Lancet*	10.1016/S0140‐6736(17)31510‐6	487
4	Priotto et al. [20]	Nifurtimox‐Eflornithine Combination Therapy for Second‐Stage African *Trypanosoma brucei gambiense* Trypanosomiasis: A Multicentre, Randomised, Phase III, Non‐Inferiority Trial	*The Lancet*	10.1016/S0140‐6736(09)61117‐X	420
5	Masiga et al. [21]	Sensitive Detection of Trypanosomes in Tsetse Flies by DNA Amplification	*International Journal for Parasitology*	10.1016/0020‐N(92)90047‐O	333
6	Franco et al. [22]	Epidemiology of Human African Trypanosomiasis	*Clinical Epidemiology*	10.2147/CLEP.S39728	318
7	Simarro et al. [23]	The Atlas of Human African Trypanosomiasis: A Contribution to Global Mapping of Neglected Tropical Diseases	*International Journal of Health Geographics*	10.1186/1476‐072X‐9‐57	283
8	Chappuis et al. [24]	Options for Field Diagnosis of Human African Trypanosomiasis	*Clinical Microbiology Reviews*	10.1128/CMR.18.1.133‐146.2005	272
9	Simarro et al. [25]	The Human African Trypanosomiasis Control and Surveillance Program of the World Health Organization 2000‐2009: The Way Forward	*PLoS Neglected Tropical Diseases*	10.1371/journal.pntd.0001007	271
10	Greenbaum et al. [26]	Synthesis and Structure‐Activity Relationships of Parasiticidal Thiosemicarbazone Cysteine Protease Inhibitors Against *Plasmodium falciparum*, *Trypanosoma brucei*, and *Trypanosoma cruzi*	*Journal of Medicinal Chemistry*	10.1021/jm030549j	268

### 3.6. Treemap and Top Keywords Analysis

**Figure 7:**
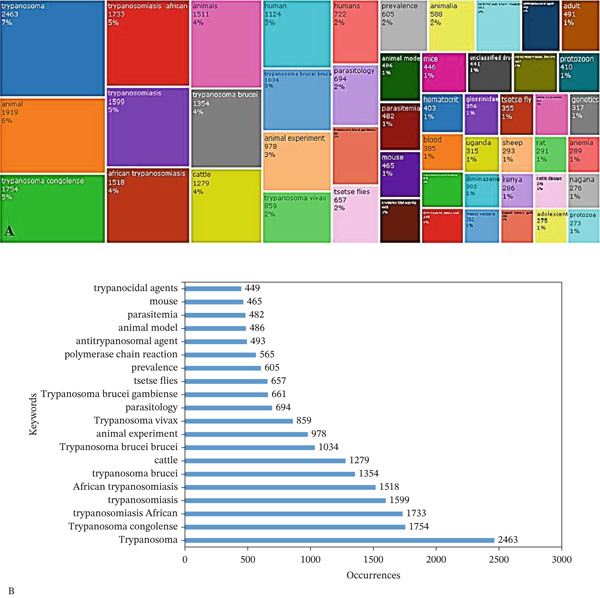
(A) Tree map based on all keywords in trypanosomiasis research in Africa and (B) most relevant keywords in trypanosomiasis research in Africa.

Figure 7A presents a tree map based on all keywords in trypanosomiasis research. Out of all the keywords, *Trypanosoma* made up 7% of keywords, appearing 2463 times, whereas animal was 6% of all keywords with 1919 appearances. Others are *Trypanosoma congolense*, *African trypanosomiasis,* and trypanosomiasis, making up 5% each of all keywords.

Figure 7B also shows the most relevant keywords in research on trypanosomiasis based on occurrence. The result shows that *Trypanosoma* was the most relevant keyword, occurring 2463 times. Additionally, *Trypanosoma congolense*, *trypanosomiasis African*, *African trypanosomiasis*, *Trypanosoma cruzi*, cattle and *Trypanosoma brucei brucei* all occurred more than 1000 times. Keywords like *animal experiment*, *Trypanosoma vivax*, and *parasitology* occurred 978, 859 and 694 times, respectively.

### 3.7. Thematic Map and Thematic Evolution

**Figure 8:**
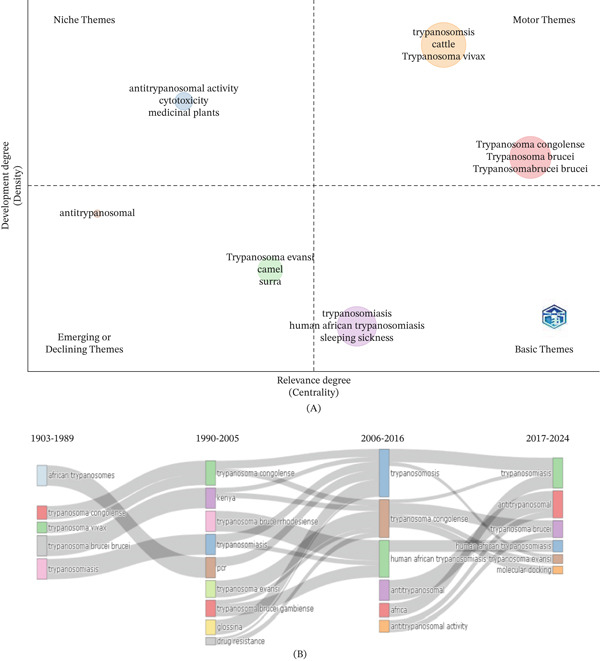
(A) Thematic map from author′s keywords in trypanosomiasis research in Africa and (B) thematic evolution based on author′s keywords in trypanosomiasis research in Africa.

Figure 8A presents the thematic map from the author′s keywords in trypanosomiasis research. This map details the degree of development (density) and relevance (centrality) of keywords explored in the research area. The map divides the research themes into two factors, which are centrality and density. Centrality shows the relevance of work in a specific theme, and density represents the development of a particular theme. The map is segmented into four quadrants, including motor theme (upper right), niche theme (upper left), emerging or declining theme (lower left), and basic theme (lower right) based on the degree of centrality (relevance) and density (development). In this research, *trypanosomosis*, *cattle*, *T. vivax*, *Trypanosoma brucei*, and *Trypanosoma brucei brucei* were the keywords defined in the motor themes of the thematic map, which is situated in the upper right quadrant. This indicated the high importance, occurrence, and immense relevance of these keywords. In the niche theme (upper left quadrant), *antitrypanosomal activity*, *cytotoxicity*, and *medicinal plants* are the keywords present. This theme is recognized for its high level of development, but it has low centrality because of its limited relevance. The emerging or declining theme consisted of *Trypanosoma evansi*, *camel, surra*, and antitrypanosomal keywords. Finally, *trypanosomiasis, human African trypanosomiasis*, and *sleeping sickness* are keywords in the basic themes located in the lower right quadrant, indicating that these keywords are of high relevance (centrality) with low development (density).

Figure 8B provides the thematic evolution based on author′s keywords in research on trypanosomiasis. Between 1903 and 1989, *African trypanosomiasis*, *T. congolense, T. vivax, Trypanosoma brucei brucei*, and *trypanosomiasis* were the dominant themes. From the period of 1990 to 2005, *T. congolense*, *Kenya*, *Trypanosoma brucei rhodesiense*, *trypanosomiasis*, *PCR*, *T. evansi, T. brucei, gambiense, Glossina*, and *drug resistance* became the most dominant themes. However, from 2006 up to 2016, the most dominant author′s keywords were *trypanosomiasis*, *T. congolense, human African trypanosomiasis*, antitrypanosomal, *Africa*, and *antitrypanosomal activity*. After 2016, *trypanosomiasis*, *antitrypanosomal*, *Trypanosoma brucei*, *human African trypanosomiasis*, *T. evansi*, and *molecular docking* became the most prevalent author′s keywords.

### 3.8. Keywords Co‐Occurrence Network

**Figure 9:**
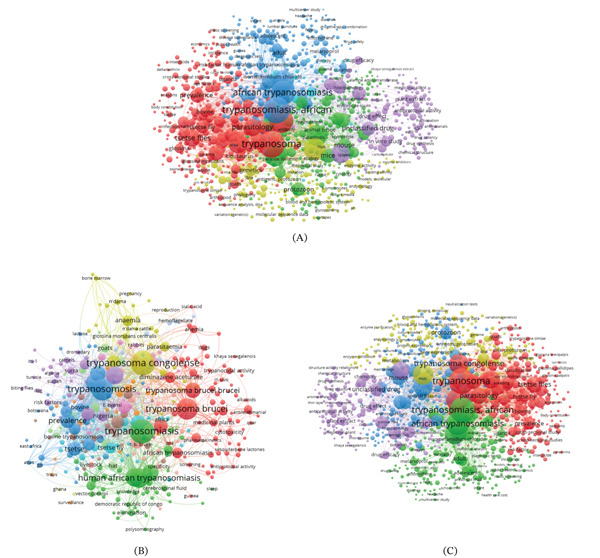
(A) All keywords co‐occurrence networks in trypanosomiasis research in Africa, (B) author′s keywords co‐occurrence network in trypanosomiasis research in Africa, and (C) index keywords co‐occurrence network in trypanosomiasis research in Africa.

Figure 9A presents the co‐occurrence network of all keywords in trypanosomiasis research in Africa, constructed using a minimum threshold of five keyword occurrences and a full counting method. The five distinctive clusters are each represented by a different color from the resulting map. Individual keywords are represented by the nodes, and the size of each node indicates the number of publications that contain the keyword in question or the frequency of the keywords′ co‐occurrence. The visualization′s density establishes the distance between two keywords; the higher the density, the closer the nodes are to each other.

Cluster 1 was the largest cluster, represented in red color, with *Trypanosoma*, *tsetse fly*, *parasitology*, *prevalence*, and *bovine* as the most occurring keywords. Cluster 2 denoted in blue color has keywords like *African trypanosomiasis, isometamidium chloride*, and *disease surveillance* as the most prevalent. The green cluster (3) captures keywords like *protozoon*, *parasite isolation*, and *animal tissue* as the most occurring. The yellow cluster (4) includes keywords like *genetics*, *enzyme activity*, *phylogeny*, and *DNA*. Lastly, Cluster 5 (purple) encompasses keywords such as *unclassified*, *in vitro study*, *drug effect,* and *histopathology*.

A primary focus on the parasite, its vectors, and the primary host is highlighted by Cluster 1. Cluster 2 suggests a focus on the disease, monitoring techniques, and drug therapies. Research on parasites, predilection sites, and the causes and course of diseases is reflected in Cluster 3. Cluster 4 emphasizes the importance of molecular tools, diagnostic processes, and drug testing. In contrast, Cluster 5 emphasizes the significance of in vitro evaluation, drug effects, and histopathological studies in the field. Collectively, the multidisciplinary nature of research on trypanosomiasis is demonstrated by these clusters, which cover basic parasitology, diagnostics, treatment, epidemiology, and veterinary science.

Figure 9B presents the author′s keyword co‐occurrence network in trypanosomiasis research in Africa. By providing valuable insight into the topic structure and development of research in this area, this network, centered on keywords that authors directly assign, provides a carefully chosen reflection of study priorities and framing. *Trypanosoma brucei, Trypanosoma brucei brucei*, *cytotoxicity*, *medicinal herbs*, and *antiprotozoal activities* are keywords of the red cluster. *Trypanosoma congolense, trypanosomosis*, *prevalence*, and *bovine trypanosomosis* are part of the main keywords of the blue cluster. The green cluster focuses on *vector control*, *tsetse fly*, *elimination*, and *human African trypanosomiasis*. Keywords like *anemia*, *parasitemia*, *Glossina morsitans*, and *n′dama cattle* are included in the yellow cluster. *Camels*, *Sudan*, *Surra*, *biting flies*, and *risk factors* are all found in the purple cluster. *Diminazene aceturate*, *T. evansi*, and treatment‐related terms are included in the smaller orange cluster. The brown cluster encompasses keywords like *livestock*, *bovine*, *trypanosomosis,* indicating a focus on the animal host and the disease.

Indexing databases, such as Scopus and Web of Science, create and standardize index keywords that account for spelling, synonyms, and plural forms [27, 28]. This gives a more comprehensive and organized thematic overview of a subject of study and sheds light on the common terminology used in scientific papers. The index keywords analysis resulted in five distinct clusters as illustrated in Figure 9C. Cluster 1 (red) is the largest cluster and comprises keywords such as *tsetse fly*, *Glossina*, and *Glossina pallidipes* centering on vector biology and ecology. Cluster 2 (blue) highlights experimental and animal models in African trypanosomiasis research with indexed keywords such as *mouse*, *rattus*, and *animal tissue*. Cluster 3 (green) has keywords including *African trypanosomiasis*, *trypanosomiasis*, and *African*, with a focus on epidemiology and public health. Cluster 4 (yellow) focuses on molecular biology and biochemistry and has keywords such as *protein expression*, *gene expression*, and *glycoproteins*. Cluster 5 (purple) has keywords such as *antiprotozoal activity*, *drug efficacy*, and *drug effect*, highlighting drug discovery and pharmacology. Keywords like *Trypanosoma, parasitology, protozoon*, and *Trypanosoma congolense* function as bridging terms, connecting across all five broader clusters.

### 3.9. Country and Authors′ Collaboration Network

**Figure 10:**
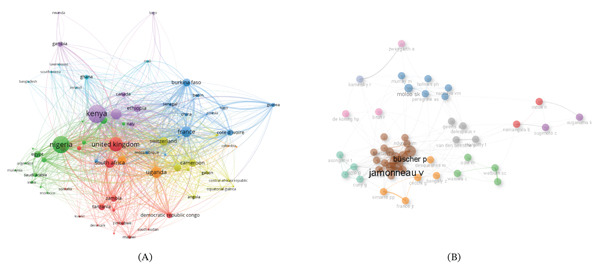
(A) Network of the coauthorship countries and (B) authors′ collaboration network.

Figure 10A illustrates the network of the coauthorship countries in African trypanosomiasis research. The network of coauthorship countries analysis is conducted to identify collaboration patterns and research impact or connection dynamics between researchers or authors from different countries in a particular field, thereby promoting knowledge exchange across borders, building research communities, providing insights for research policy‐making, and funding collaboration and international collaboration initiatives. Setting the threshold at a minimum of five documents, seven (7) distinct clusters were identified using a network visualization pattern for clustering, demonstrating varied collaboration dynamics. Kenya, Nigeria, the United Kingdom, Uganda, France, Switzerland, and Ghana represent key clusters in the map. Each cluster depicts significant intercontinental collaborations as evident with partnerships among countries from different continents. This trend illustrates how trypanosomiasis research is conducted worldwide and how crucial international collaborations are to the field′s advancement.

The authors′ collaboration network is shown in Figure 10B, emphasizing the institutional and interpersonal ties that propel research output. The brown cluster emerged as the dominant cluster and is led by Buscher P. and Jamonneau V., who were also the most impactful authors in the field. Several dense clusters that individually represent research teams or organizations and are frequently anchored by eminent universities or prolific authors define the network. Some authors facilitate interdisciplinary and interinstitutional collaboration by acting as linkages between clusters. This structure illustrates the collaborative nature of the trypanosomiasis research community, where knowledge and resources are shared to address complex scientific and public health challenges.

## 4. Discussion

### 4.1. General Overview and Publication by Year

Bibliometrics has quickly emerged as a distinct field in recent years. It combines mathematical principles, statistical analysis, and logical reasoning to examine various facets of published works [7, 29]. In this study, bibliometric techniques were used to examine the research output and trends in trypanosomiasis research in Africa. There has been a considerable increase in research output related to this field (Figure 3A), indicating that new concepts, investigations, and deductions are introduced annually; this also suggests the dynamic and ever‐evolving nature of this research area. Similarly, the international coauthorships percentage (47.94; Figure 2) highlights significant global networking in generating scientific output on African trypanosomiasis research. This shows that international collaboration is responsible for almost half of all publications in the research area, thus increasing the variety of perspectives, skills, and funding invested in its research. This further indicates strong global collaboration, access to broader research infrastructure, and a shared international interest in combating this neglected tropical disease.

From the analysis of annual scientific production, it can be deduced that there has been an increasing research interest in the field, leading to growth in publications. The highest average total citations of articles on trypanosomiasis research per year were observed within the periods of increasing scientific publication (Figure 3A,B). Despite the decline in the number of cases from approximately 25,800 in 2000 to about 675 in 2023 [30] within Africa, research remains active. The literature shows a notable rise in publications and reviews on African trypanosomiasis in the past few years (Figure 3A). Specifically, across Africa, there is a clear rise in mapping, prevalence studies, resistance analysis, and re‐emergence reports since 2020. The country‐level analysis further supports this trend. In Nigeria, there is a post‐2000 studies surge, although there are reports of re‐emergence and case reports in Ethiopia, with several West/Central Africa nations publishing atlases and control data. The number and variety of publications, including meta‐analyses, surveillance reports, field studies, and drug resistance investigations, have significantly expanded recently [31].

Thompson′s analysis of 5139 articles revealed a significant growth, with publication doubling approximately every 39.5 years. He noted that “epidemic” peaks occurred in 1910–1914 and 1979–1985, demonstrating surges in scientific interest during this period [9]. A systematic review by Okello et al. [32] on sub‐Saharan AAT identified 304 articles published between 2000 and 2021, with the bulk focused on livestock (192 studies), vector prevalence (44), and resistance analysis (30). This highlights a substantial body of recent research.

### 4.2. Country Performance and Collaborations

Analysis of country performance shows that Kenya and Nigeria are the leading nations in research on trypanosomiasis in terms of the number of research publications. A number of reasons could potentially explain this. Firstly, both countries are highly endemic for AAT and have had a historical burden of HAT. The disease affects livestock productivity and human health, creating a strong national need for scientific research and control measures [33, 34]. In addition, both countries host dedicated research centers for trypanosomiasis. In Kenya, there is the Trypanosomiasis Research Centre, the Kenyan Medical Research Institute (KEMRI), the International Livestock Research Institute (ILRI), and the International Centre for Insect Physiology and Ecology (ICIPE), among others. Similarly, in Nigeria, there are the Nigerian Institute for Trypanosomiasis Research (NITR) and the National Veterinary Research Institute (NVRI). These institutions promote high‐volume scientific output and offer platforms for postgraduate and international collaborative research on trypanosomiasis [35]. Lastly, both Kenya and Nigeria have high international coauthorship rates in trypanosomiasis publications. Collaborations with institutions like WHO/TDR, FAO/IAEA, Wellcome Trust, ILRI, and National Institute of Health (NIH) contribute to research funding, capacity building, and dissemination [35]. Interestingly, the European trio of the United Kingdom, France, and Belgium are the other top contributors to research on trypanosomiasis in Africa. This suggests that African trypanosomiasis research is not just restricted to endemic regions but has a global interest, as Switzerland, Japan, and the United States constitute the last three of the Top 10 country contributors to research in the field (Figure 4A).

Furthermore, the United Kingdom, France, Belgium, and Switzerland make up the Top 4 of the most cited countries in this study. Aside from their long‐standing historical and colonial ties with Africa [36], each of these countries hosts prestigious institutions that have played a central role in trypanosomiasis research, contributing foundational knowledge, advanced diagnostics, and innovative control strategies. These institutions conduct cutting‐edge research and field trials and publish in high‐impact journals, resulting in high citation rates [25, 33, 37]. Furthermore, these countries actively contribute to the WHO′s strategic goals for the elimination of HAT as a public health problem [34]. Kenya and Nigeria rank fifth and sixth, respectively, in terms of the most cited countries of scientific publications in the research area.

Meanwhile, the topmost relevant country of publications based on the corresponding author was Nigeria, with 363 articles. Nigeria has 82.6% SCP and 17.4% MCP. The United Kingdom, France, Kenya, and Belgium follow with 207, 142, 136, and 134 articles, respectively. Among the African countries represented in the Top 10 (Table 1), Cameroon (ranked 9th) and South Africa (ranked 6th) had the highest percentage of MCP (83.8% and 54.1%), respectively. This indicates more intercountry collaboration. All the non‐African nations had more MCP compared with SCP, indicating more intercountry than intracountry collaborations. This is because the study focused specifically on Africa; therefore, studies involving non‐African countries are virtually studies done in collaboration with an African country. Meanwhile, there is a need for African nations to engage in more international collaborations to completely address this public health threat.

### 4.3. Affiliations and Sources′ Contributions

Authors typically have ties to organizations or institutions where they carry out their research. In order to assess research trends and institutional output within a certain body of knowledge, affiliation is also helpful in bibliometric analyses. The most relevant affiliations of scientific publications in the research area identified from this analysis included Ahmadu Bello University (ABU) (Nigeria), Institute of Tropical Medicine (Belgium), University of Nigeria (Nigeria), University of Glasgow (United Kingdom), Makerere University (Uganda), and the University of Edinburgh (United Kingdom), among others. These institutions are also located in the most relevant countries, where African trypanosomiasis research output is significant. A number of historical, geographical, scientific, and collaborative factors have contributed to the prominence of these institutions in research on trypanosomiasis in Africa. ABU is situated in northern Nigeria, which is a hotspot for both HAT and AAT, making Nigeria one of the most endemic countries for both diseases. As the home of the Trypanosomiasis Research Unit and the Faculty of Veterinary Medicine, ABU serves as a hub for surveillance and research [38]. The Institute of Tropical Medicine has a long colonial and postcolonial history of parasitology research in sub‐Saharan Africa, particularly in the DRC, which has historically had the largest HAT burden. It is a global leader in trypanosomiasis drug research, control policies, and diagnostics, particularly through partnerships with national control programs and the WHO [19, 39]. Similarly, the University of Nigeria, Nsukka serves as a regional hub for trypanosomiasis surveillance and research, demonstrated through field diagnosis in horses and exploration of drug resistance via ethnobotanical studies [40, 41]. The University of Glasgow has been a major player in parasitology and vector biology, and it has worked closely with African institutions, especially on immunopathogenesis, trypanosome genomes, and tsetse fly biology. Kinetoplastid illnesses are the main focus of Glasgow′s Wellcome Centre for Integrative Parasitology [42], whereas Uganda has been a key focus of *gambiense* and *rhodesiense* HAT research and control programs. Makerere′s College of Health Sciences and College of Veterinary Medicine work closely with the Ministry of Health and WHO on surveillance and elimination programs. The university also contributes to drug trials and health systems research [43]. Finally, as the home to the Centre for Immunity, Infection, and Evolution, the University of Edinburgh is a hub for host–pathogen interaction studies, immunogenomics, and the evolution of virulence in trypanosomes. It leads computational biology and modeling collaborations with African partners [44].

Journals and conference proceedings, which are often the sources of scientific knowledge, are equally pertinent contributions to the creative assessment of research impact. *Acta Tropica*, *Annals of Tropical Medicine and Parasitology*, *Veterinary Parasitology*, *Transaction of the Royal Society of Tropical Medicine and Hygiene*, and *PLOS Neglected Tropical Diseases* are the most relevant sources, in which trypanosomiasis‐related research in Africa was published (Figure 5B). The status of these journals as major publication hubs for trypanosomiasis‐related research in Africa is grounded in their editorial scope, historical focus on tropical diseases, and international recognition. Each of these journals consistently publishes high‐impact, peer‐reviewed studies on epidemiology, diagnostics, parasitology, vector biology, and control of diseases endemic to Africa, particularly trypanosomiasis. Similarly, with regard to the source impact of scientific publications in the field (Figure 5C), *PLOS Neglected Tropical Diseases* came first in the list of the Top 10 sources, followed by *Acta Tropica*, *Veterinary Parasitology, Parasites and Vectors,* and *Parasitology*. *Parasite Immunology* and *Experimental Parasitology* were the last two among the Top 10 sources with the highest impact on scientific publications in the research area (Figure 5C). This data regarding the source impact of scientific publications in the field further provides critical insights into the visibility, relevance, and influence of journals within the academic and research ecosystem addressing this neglected tropical disease. The source impact distribution among these top journals reflects a diverse research ecosystem, including open‐access and public health–oriented (e.g., *PLOS Neglected Tropical Diseases*), classical and experimental (e.g., *Parasitology* and *Experimental Parasitology*), and vector‐ and veterinary‐focused (e.g., *Veterinary Parasitology*, and *Parasites & Vectors*) repositories. This diversity demonstrates that research in the field spans multiple disciplines, each finding its niche in specific high‐impact journals. These journals drive the advancement of knowledge and the development of effective strategies to combat one of Africa′s most persistent parasitic diseases.

### 4.4. Author′s Productivity and Impact

A total of 8849 authors contributing to African trypanosomiasis research reflect a significant level of scholarly interest and involvement in addressing this neglected tropical disease (Figure 2). This number underscores the disease′s continued relevance in both human and animal health across the continent [37, 44]. However, a deeper analysis reveals a disparity in authorship patterns. Notably, most publications from African nations tend to be SCP. This pattern may indicate limitations in collaborative research infrastructure, institutional isolation, and restricted access to international funding within many African institutions. In contrast, non‐African–led studies often benefit from structured consortia, cross‐border funding mechanisms, and multidisciplinary teams [45, 46]. More comprehensive research outputs are usually the products of these collaborative structures, which are often published in high‐impact journals. This discrepancy suggests a need for increased support for intra‐African and international collaborations that prioritize equitable partnerships. Empowering African researchers through coauthorship inclusion, partnerships, and capacity‐building initiatives is vital for advancing locally led solutions to trypanosomiasis. Fostering research equity is critical to enhancing both the quality and visibility of African scholarship in global health discourse.

A core group of researchers driving knowledge production in the field of African trypanosomiasis is highlighted by the authorship analysis in Figure 6A, which identifies the most prolific contributors to this field of study. Interestingly, Buscher, P. comes in first with 110 publications, highlighting his pivotal role in improving epidemiology, diagnosis, and control methods for *Trypanosoma* spp. Buscher′s substantial production, frequently produced through partnerships at the Institute of Tropical Medicine in Belgium, demonstrates a persistent global dedication to the fight against African trypanosomiasis [19]. Moloo, S.K. comes in second with 107 publications, many of which concentrate on field monitoring and diagnostic tool optimization, especially in endemic areas. With 93 papers, Jamonneau, V. has made a substantial contribution to the study of immunopathogenesis and the creation of treatments. Notable African‐based researcher Matovu, E. of Uganda (75 publications), is known for his research on trypano‐tolerance and medication resistance, exhibiting strong local leadership in scientific production [47]. With 73 articles, Welburn, S.C. has been instrumental in socioecological research and One Health (OH) methods, connecting the dynamics of trypanosomiasis in humans and animals. These Top 5 authors exemplify the blend of African and international expertise shaping the current landscape of trypanosomiasis research, and their output reinforces the importance of long‐term commitment and collaboration. The remaining authors among the Top 10 have collectively contributed extensively to African trypanosomiasis research in aspects such as vector biology, ecology, and immunological and molecular studies.

Figure 6B illustrates the publication timeline of the Top 10 most productive authors in African trypanosomiasis research between 1973 and 2024, marking a critical phase of sustained and evolving scientific engagement. The data highlight distinct temporal patterns among these leading researchers, reflecting both generational shifts and the progression of scientific inquiry over time. Buscher, P. emerged as a key contributor from 1999 to 2024, with consistent productivity over 25 years, coinciding with advances in molecular diagnostics and integrated control strategies. His sustained output aligns with the period of intensified global efforts to eliminate *T*. *b*. *gambiense*, including WHO‐led initiatives. Moloo, S.K., on the other hand, contributed a great deal between 1973 and 2003, which is an earlier wave of fundamental research, especially in the construction of early vector monitoring systems and field diagnostics. Critical foundations for subsequent advancements were established by this early stage. From 1998 to 2024, Jamonneau, V. made substantial contributions to immunology and therapeutic interventions, especially in the postgenomic period of trypanosome research. Comparably, from 1997 to 2024, Matovu, E. and Welburn, S.C. both maintained strong publication paths, demonstrating sustained interest in topics including medication resistance, host–parasite interactions, and OH frameworks. The robustness and breadth of African trypanosomiasis research are enhanced by these overlapping timeframes, which point to a generational convergence of expertise with long‐lasting partnerships and changing thematic focus.

Table 2 highlights the top authors with the highest impact in the field based on their h‐index, which reflects both productivity and citation impact. Buscher, P., with the highest *h*‐index (35), has significantly advanced the diagnosis, epidemiology, and control of HAT, influencing WHO guidelines and diagnostic protocols [19, 48]. Jamonneau, V. (*h*‐index 34) has made significant contributions to the field of molecular parasitology, focusing on asymptomatic carriers, disease staging, and biomarker development for *T. b. gambiense* [49, 50]. Lejon, V. (*h*‐index 30) has enhanced staging tools and deepened understanding of CNS involvement through cerebrospinal fluid biomarker research [51]. Solano, P.′s (*h*‐index 29) research has advanced knowledge of transmission dynamics, vector biology, and population genetics, aiding control strategies in West and Central Africa [52]. Welburn, S.C. (*h*‐index 29) promoted a “One Health” approach, examining zoonotic interfaces and socioecological factors shaping disease control [53]. These authors, among others, shaped the scientific foundation of trypanosomiasis control, linking academic rigor with practical disease elimination efforts across the continent.

### 4.5. Analysis of the Most Cited Documents

Original research articles predominate in Table 3, which lists the most cited academic publications that have had a major impact on trypanosomiasis research in Africa. The work by Hirumi and Hirumi [17] is the most cited. A novel technique for the continuous in vitro growth of *T. brucei* bloodstream forms without feeder layers was presented in this study. This innovation has enhanced experimental reproducibility and accelerated drug discovery and molecular studies. The second on the list is Murray et al. [18], which revealed that trypanosome infections cause immunosuppression and anemia in cattle, laying the foundation for vaccine development and control strategies for AAT. In third place is Buscher et al. [19], which presented updated diagnostic guidelines for HAT, emphasizing integrated parasitological, serological, and molecular tools crucial for surveillance and clinical decision‐making, especially in postelimination contexts. The fourth most cited paper is Priotto et al. [20], which validated the use of nifurtimox–eflornithine combination therapy (NECT) for second‐stage *T. b. gambiense* HAT, which is now a WHO‐endorsed standard treatment. Masiga et al. [21] emerged fifth on the list and developed species‐specific DNA probes, enabling precise identification of African trypanosomes, a vital advancement for diagnostics and epidemiological studies. The first five of the 10 most cited documents on African trypanosomiasis research address key areas such as diagnostics, host–pathogen interactions, treatment, and parasite biology. They continue to underpin translational and policy‐driven research aimed at the control and eventual elimination of trypanosomiasis across the African continent.

The remainder of the Top 10 most cited documents present several other influential studies that have significantly transformed African trypanosomiasis research, with a focus on epidemiology, diagnostics, surveillance, and drug discovery. For instance, Franco et al. [22] provided a critical overview of HAT distribution, burden, and epidemiological trends, emphasizing the shift toward elimination and the challenge of asymptomatic carriers. Simarro et al. [23] introduced the Atlas of HAT, a geo‐referenced database of cases across Africa that has become vital for disease modeling and targeted control. Chappuis et al. [24] assessed field diagnostic tools, including the card agglutination test for trypanosomiasis (CATT) and molecular methods, influencing WHO diagnostic guidelines. Another pivotal study by Simarro et al. [25] evaluated WHO′s HAT control program (2000–2009), documenting incidence reduction and advocating for integration into national health systems and sustained funding. In the area of drug development, Greenbaum et al. [26] demonstrated the efficacy of thiosemicarbazone‐based cysteine protease inhibitors against *T. brucei*, *T. cruzi*, and *Plasmodium falciparum*, highlighting the potential of multipathogen drug design. These multidisciplinary studies continue to inform both scientific understanding and practical strategies for trypanosomiasis control and elimination in Africa.

A general overview of the most cited documents on trypanosomiasis research in Africa reveals that the highest impact studies are those that address practical public health needs, particularly in areas such as diagnostics, epidemiological surveillance, and disease control strategies. Foundational works on parasite cultivation and diagnostic tools, like those by Hirumi and Hirumi [17] and Chappuis et al. [24], reflect the importance of laboratory innovation in the field; similarly, publications by Franco et al. [22] and Simarro et al. [23, 25] emphasize the significance of epidemiological mapping and WHO‐aligned surveillance in directing elimination efforts. The importance of collaborative, interdisciplinary studies highlights the value of the OH approach. Meanwhile, the relatively low representation of drug discovery research among the top‐cited works suggests a potential gap in therapeutic innovation, and the focus on *T*. *b*. *gambiense* suggests a regional and thematic bias toward West and Central Africa. Generally, the citation trends emphasize that research with direct policy relevance, global applicability, and a strong implementation focus tends to achieve the highest scientific impact.

### 4.6. Treemap and Top Keywords Analysis

Figure 7A presents a treemap visualization of all keywords used in research on trypanosomiasis in Africa, highlighting the thematic focus and research trends within this field. The largest segments, such as *Trypanosoma* (7%), *animal* (6%), *trypanosomiasis* (5%), and species‐specific terms like *Trypanosoma congolense* (5%) and *Trypanosoma brucei (4%),* reflect a strong emphasis on the etiologies and their relevance to both animal and human hosts. Keywords such as *animals*, *cattle*, *human*, and *animal experiment* highlight the disease′s zoonotic and veterinary significance, which is in line with the OH strategy in endemic areas [22, 25]. Geographical keywords such as *Uganda* and *Kenya*, as well as terms like *tsetse flies* and *Glossinidae*, suggest a focus on vector ecology and high‐burden regions [54, 55]. Other keywords used are essential for illness monitoring and treatment response; they demonstrate that research also encompasses clinical and diagnostic elements. These include *parasitemia*, *hematocrit*, and *anemia* [48, 56]. Additionally, words like *diminazene*, which are used to highlight therapeutic treatments, reflect ongoing research into trypanocidal medications and resistance [57]. The presence of keywords like *genetics*, *protozoa*, and different laboratory animal models (such as *mouse*, *mice*, and *rat*) further suggests a keen interest in molecular biology and experimental study. The treemap depicts an extensive and interdisciplinary research environment that addresses trypanosomiasis epidemiology, pathology, diagnostics, therapy, and control in Africa.

Figure 7B illustrates the most relevant keywords in the study based on their frequency of occurrence, offering insights into dominant themes and research priorities within the field. The keyword *Trypanosoma* appeared most frequently, indicating the centrality of the parasite genus in African trypanosomiasis research. The emphasis on species‐specific and regional disease burden is reflected in other frequently used topics, such as *T*. *congolense, trypanosomiasis African*, *trypanosomiasis*, and *African trypanosomiasis*. The importance of *T*. *brucei* and its subspecies, especially *T. b. brucei*, highlights the research focus of infectious strains that affect humans. Furthermore, laboratory models like *mouse*, as well as host‐related terms like *cattle* and *animal experiment*, are strongly represented, indicating a OH strategy that combines veterinary and biomedical sciences [22, 25]. The prominence of research instruments and subjects, including *polymerase chain reaction*, *parasitemia*, *tsetse flies*, and *antitrypanosomal agents,* suggests a concentration on diagnosis, transmission, and therapeutic approaches. The result as a whole depicts a targeted and interdisciplinary research environment addressing the intricate pathogenesis, epidemiology, and control of trypanosomiasis in Africa.

### 4.7. Thematic Map and Thematic Evolution

A thematic map based on the author′s keywords is shown in Figure 8A. The themes are categorized according to their development degree (density) and relevance degree (centrality). *Trypanosomosis*, *cattle*, and *T. vivax* are among the well‐developed and important topics found in the upper right quadrant (motor themes), demonstrating their crucial relevance in contemporary research and applications, especially in veterinary contexts. Key agents of AAT, *T. congolense*, *T. brucei*, and *T. b. brucei*, are also found in this region, highlighting their significance for cattle health. Specialized but less important themes including *antitrypanosomal activity*, *cytotoxicity*, and *medicinal plants* are included in the upper left quadrant (niche themes). Despite their development, these themes have little wider impact on the field. On the other hand, fundamental but underdeveloped themes like *human African trypanosomiasis*, *trypanosomiasis*, and *sleeping sickness* are located in the lower right quadrant (basic themes), underscoring their importance while also pointing to the necessity of additional scientific investigation and integration. Lastly, themes like *T. evansi, camel*, *surra*, and *antitrypanosomal*, which may indicate new study directions or diminishing interest, are seen in the lower left quadrant (developing or declining themes). The current research goals, gaps, and future directions in African trypanosomiasis investigations are well visualized by this theme structure.

The thematic evolution of the research field from 1903 to 2024, based on the author′s keywords, highlights a shift in research priorities and thematic foci across four distinct periods (Figure 8B). In the earliest period (1903–1989), themes such as *African trypanosomes*, *T*. *congolense*, *T. vivax*, *T. b*. *brucei*, and *trypanosomiasis,* which are foundational themes in African trypanosomiasis research, dominated the literature. Between 1990 and 2005, there was a diversification of themes with emerging interest in *PCR diagnostics*, geographical contexts like *Kenya*, and subspecies such as *T. b*. *rhodesiense*, *T. b*. *gambiense*, and *T. evansi*, as well as concerns related to *drug resistance* and *Glossina*. A growing interest in therapeutic development and the significance of human disease was indicated by the consolidation of research from 2006 to 2016 around broader themes such as *trypanosomosis*, *human African trypanosomiasis,* and *antitrypanosomal activity* [22]. Newer topics, including *molecular docking* and a greater emphasis on *antitrypanosomal drugs* in the most recent phase (2017–2024), demonstrate how computational biology and drug discovery techniques are being integrated in the area [3]. *T. congolense, T*. *brucei,* and trypanosomiasis are examples of persistent themes that show the disease′s ongoing significance in African research contexts. This development demonstrates how this research field has moved away from geographic and taxonomic descriptions and toward contemporary, multidisciplinary methods that deal with molecular biology, diagnostics, and treatments.

### 4.8. Keyword Co‐Occurrence Network

A co‐occurrence network of all keywords is shown in Figure 9A, which illustrates theme clusters and links across different fields of study. *Trypanosoma, African trypanosomiasis,* and *parasitology* are the main and most noticeable keywords that act as interconnecting links between several fields of study. The red cluster, which includes keywords such as *tsetse flies, Glossina, bovine, infection rates, and prevalence*, is primarily focused on vector‐related research, underscoring the ongoing significance of epidemiological and entomological investigations in livestock and public health. *Human African trypanosomiasis, melarsoprol, eflornithine, medication therapy,* and *disease surveillance* are all included in the blue cluster, which is focused on clinical and therapeutic themes and reflects medical interventions and clinical research goals [22]. Meanwhile, keywords like *mouse, mice, animal tissue, histopathology,* and *unclassified drug* in the green cluster indicate focus on experimental models and pathogenesis. This underscores the value of both in vivo and laboratory‐based research in comprehending host–parasite interactions and drug evaluation. The yellow cluster indicates molecular and genetic research, referring to contemporary developments in molecular parasitology and immunogenetics through the use of terms like *protozoon, genetics, phylogeny, sequence analysis, enzyme activity,* and *epitopes.* Finally, with keywords like *plant extract, medicinal plant, antiprotozoal activity, drug efficacy,* and *chemical structure,* the purple cluster represents phytotherapy and pharmaceutical sciences and indicates a growing interest in natural products and bioassay‐guided drug discovery for antitrypanosomal agents [3]. With strong connections between clinical interventions, epidemiology, experimental models, molecular biology, and natural product research, the network generally demonstrates a multidisciplinary structure of the research domain. This structure reflects changing tactics to combat African trypanosomiasis in both humans and animals.

Furthermore, the author keywords′ co‐occurrence network highlighted the thematic structure and interrelated domains within the field (Figure 9B). Central nodes such as *trypanosomiasis, T*. *congolense, T*. *brucei,* and *human African trypanosomiasis* dominate the network, indicating their centrality and high frequency in the research discourse. The color‐coded clusters reflect thematic groupings: For instance, the red cluster with terms such as *medicinal plants, cytotoxicity, pharmacokinetics,* and *antiprotozoal activity* centers on pharmacological research and indicates the growing interest in bioactive substances such as alkaloids and sesquiterpene lactones. The blue cluster focuses on veterinary trypanosomiasis and its epidemiology in animals with keywords such as *prevalence, bovine trypanosomosis,* and *tsetse* indicating regional studies in Nigeria, Botswana, and East Africa [23]. The terms *elimination, vector control,* and *surveillance* in the green cluster center on public health dimensions, in regions like the DRC, underlining efforts to control HAT [22]. The yellow cluster′s keywords like *anemia*, *parasitemia*, and *reproduction* point to immunopathological and reproductive research using animal models. *Camel, Sudan, Surra*, *biting flies*, and *risk factors* are all found in the purple cluster, reflecting region, animal host, disease, and vector. *Diminazene aceturate, T. evansi,* and treatment‐related terms are included in the smaller orange cluster. Finally, the brown cluster encompasses keywords like *livestock*, *bovine*, and *trypanosomosis,* indicating a focus on the animal host and the disease. These clusters provide a comprehensive overview of the several topics in this research field. This network highlights the multidisciplinary and interconnected nature of African trypanosomiasis research, ranging from molecular parasitology and drug discovery to epidemiological surveillance and disease elimination strategies.

The index keywords co‐occurrence network in African trypanosomiasis research reveals the structural and thematic organization of indexed scientific focus areas (Figure 9C). Central terms such as *Trypanosoma*, *African trypanosomiasis*, and *Trypanosoma congolense* dominate the map, highlighting their prominence in research outputs. The red cluster is deeply oriented toward vector biology, emphasizing species such as *Glossina pallidipes* and *Glossina morsitans*, along with environmental and ecological terms like *seasons, ecosystem,* and *insecticide,* reflecting integrated vector control research [33]. The green cluster concentrates on epidemiological and clinical aspects, with co‐occurring keywords like *isometamidium chloride, melarsoprol, adult, mortality,* underscoring treatment options and efficacy. The blue and yellow clusters cover molecular and immunological studies, with index keywords such as *molecular sequence data, antigens, protozoan,* and *gene expression,* signifying a strong foundation in molecular parasitology and host–pathogen interactions. On the left, the purple cluster reflects pharmacological and drug discovery efforts, where keywords like *plant extract, unclassified drug, antiprotozoal activity,* and *melarsoprol* suggest ongoing research into both synthetic and natural therapeutic compounds [57]. The visualization shows how diverse disciplines including public health, pharmacology, vector ecology, and molecular biology interconnect to form a robust, interdisciplinary research landscape addressing trypanosomiasis in Africa.

### 4.9. Country and Authors′ Collaboration Network

The coauthorship network among countries engaged in trypanosomiasis research in Africa demonstrates the global collaborative landscape underpinning this field (Figure 10A). Prominent nodes such as Kenya, Nigeria, the United Kingdom, France, Uganda, and Switzerland indicate their pivotal roles in scholarly output and international cooperation. The thick interconnecting lines, particularly between African and European countries, notably the United Kingdom, France, and Switzerland, highlight long‐standing partnerships in research, capacity building, and field surveillance programs [22, 25]. For instance, Kenya emerges as a central hub, reflecting the extensive work of research centers like the Kenya Medical Research Institute (KEMRI) and its collaborations with the UK′s Liverpool School of Tropical Medicine and the Swiss Tropical and Public Health Institute (Swiss TPH). Similarly, Nigeria′s strong ties with South Africa and the United Kingdom emphasize its growing role in regional research leadership. The widespread connections across African nations, such as between Uganda, the DRC, Cameroon, and Burkina Faso, indicate increased intra‐African scientific collaboration, a trend encouraged by international funding bodies and regional networks like AUDA‐NEPAD. This network visualization underscores how transnational partnerships are critical for advancing diagnostics, therapeutics, and vector control strategies in endemic regions.

Similarly, the authors′ collaboration network in African trypanosomiasis research illustrated clusters of researchers who frequently coauthor scientific publications (Figure 10B). Central to this network are Jamonneau, V. and Buscher, P. whose names appear prominently due to their high degree of coauthorship and collaborative influence. These authors act as key connectors among various research teams, indicating their central role in advancing the field. The brown cluster centering around them also includes collaborators such as Ndung′u, J.M. and Biteau, N., suggesting a cohesive and prolific group likely engaged in diagnostics and epidemiological studies of HAT. Other distinct clusters, such as those involving Moloo, S.K., Kaminsky, R., and Holmes, P.H. (blue), or Sugimoto, C. and Suganuma, K. (purple), indicate focused collaborations that may be regionally or thematically specialized, such as vector biology or molecular pathogenesis. The dispersion and connections between clusters imply both regional and interdisciplinary collaborations, with some authors like Namangala, B. and Odiit, M. serving as bridges between otherwise separate research groups. This network highlights the importance of collaborative research efforts in neglected tropical diseases and supports prior assertions that global partnerships are crucial in addressing complex parasitic diseases like trypanosomiasis [22, 25].

### 4.10. Trypanosomiasis Research in Africa: Way Forward

Although African trypanosomiasis has declined in overall incidence and prevalence in the last two decades, AAT remains a persistent threat in sub‐Saharan Africa, negatively impacting livestock productivity and agricultural output [32]. There is also a possibility of spillover from animals to humans due to parasite evolution and adaptation. To effectively and comprehensively address this public health threat, the following strategies are suggested [58].

#### 4.10.1. OH Approach

A OH strategy is crucial due to the zoonotic potential of this disease, especially with *T*. *b*. *rhodesiense*. It is crucial to treat illnesses in both humans and animals and to prevent them from spilling over into the environment, as shown by the centrality of human and animal trypanosomiasis in Figure 8A. Research has shown that treating cattle lowers the rates of human infections [59]. With the help of agencies like the FAO and WHO, coordinated efforts between the medical, veterinary, and ecological sectors are required [60, 61].

#### 4.10.2. Strengthening Health Systems and Capacities

It is also critical to strengthen health systems and increase research capability in endemic areas. Findings presented herein showed more SCP than MCP across most African nations (Table 1). A collaborative approach is required to address this issue. Sustainable monitoring and innovation systems are supported by investments in laboratory infrastructure, training, and regional partnerships like the Pan African Tsetse and Trypanosomiasis Eradication Campaign (PATTEC) [62].

#### 4.10.3. Improving Diagnostic and Surveillance Tools

Improving diagnostics and surveillance tools is crucial in mitigating the risks posed by African trypanosomiasis. Although molecular diagnostics such as PCR facilitate species separation and resistance monitoring (Figures 7B and 8B), advanced diagnostic methods such as LAMP‐ and CRISPR‐based assays will improve field detection [63–65]. Furthermore, hotspot mapping is further facilitated by GIS‐based surveillance [33], aligning with the identification of disease surveillance and monitoring techniques as notable themes in Figure 9A.

#### 4.10.4. Ecological and Epidemiological Research

To evaluate animal reservoirs, the effects of climate change on tsetse distribution, and new urban transmission risks, ecological studies are essential [33, 66]. These will provide insight into the risks posed by animal reservoirs (Figure 7A) and forecast the role of climate change in disease epidemiology. This research would inform national and regional policies to support community involvement in the sustainable eradication of African trypanosomiasis [25].

#### 4.10.5. Innovative Prophylactic and Therapeutic Strategies

Although oral fexinidazole represents a step forward in the therapeutic management of African trypanosomiasis, limitations remain regarding efficacy, resistance, and disease stage [67, 68], as highlighted in Figure 8B. Combination therapy and drug repurposing continue to offer promising avenues to strengthen treatment pipelines [69]. Antigenic variation remains a significant obstacle to vaccine development, as it enables parasites to evade host immune responses; however, research into conserved targets is ongoing. Innovations in vector control, particularly community‐led approaches that integrate paratransgenesis, gene editing, and the sterile insect technique (SIT), show considerable promise for sustainable control of transmission [70–72].

#### 4.10.6. Future Funding Priorities

The localization of OH programs at human–animal–environment interfaces is an eminent priority. Transdisciplinary OH initiatives that integrate veterinary, medical, ecological, and social sciences at endemic sites with cocreation with communities and adaptive governance should be funded to enhance the fight against the disease [73]. Similarly, long‐duration programmatic funding that supports cross‐institution governance, shared procurement, joint training, and transparent IP arrangements deserves sustained investment. Besides, addressing the underrepresentation of Global South–Global South collaboration and improving Africa‐led leadership in trypanosomiasis research are pertinent. Funding calls should favor African prime‐led consortia, with cofunding and researcher mobility to strengthen domestic capacities [74]. Community‐based participatory research and codesign of vector‐control tools, diagnostics, and surveillance strategies, recognizing the importance of local social‐ecological contexts for sustained uptake, should be prioritized [73].

Capacity building for transdisciplinary research literacy and ethics is also paramount. Curricula and long‐term training in transdisciplinary methods, data governance, ethics, and community engagement should be readily funded. The low centrality of *medicinal plants* in the niche theme (Figure 8A) suggests an underexplored area with potential for novel antitrypanosomal agents, warranting increased research funding and collaborative efforts between ethnobotanists and parasitologists.

Open science, data sharing, and digital infrastructure require good investment. They promote interoperable data platforms, shared protocols, and governance for cross‐sector data use. Open‐science and joint data infrastructure could enable cocreation and rapid translation [75]. Additionally, studies that integrate climate data with vector ecology, livestock management, and human health outcomes to produce climate‐resilient control strategies should be prioritized for funding, as they can deliver policy‐ready guidance for adaptation and resilience planning at regional scales [76].

Meanwhile, crucial technological focal areas requiring good funding include the development of cross‐sector surveillance platforms (human, animal, and environment) using eDNA, rapid diagnostics, and mobile data collection to improve detection and rapid response. Similarly, next‐generation diagnostics (LED‐based microscopy, LAMP, and rapid tests) and field‐appropriate diagnostic workflows to enable timely treatment and control require financial investment [77, 78]. Besides, eco‐efficient vector‐control tools, live‐bait strategies, and research on trypanotolerance genetics to reduce reliance on chemicals and sustain control efforts are other promising aspects [79, 80]. Establishment of OH innovation hubs in high‐burden regions; data infrastructure and AI for trypanosomiasis; and African‐led cross‐border trypanosomiasis consortia for regional vector surveillance, harmonized data standards, and shared modeling platforms could revolutionize the continental fight against trypanosomiasis [79, 81–83].

### 4.11. Limitations

Although this work offers important insights into African trypanosomiasis research, it has some limitations that should be taken into account to further contextualize the results presented herein. Firstly, the study only used publications that were indexed in the Scopus database. Because the focus was solely on papers sourced from the Scopus database, many other databases, including the Web of Science, Google Scholar, Dimensions, and Lens, were excluded. Although this could seem like a drawback, the Scopus database is regarded as the most reliable source for academic publications [84–88]. Moreover, restricting the analysis to English‐language publications introduces a potential bias [85]. This may favor English‐speaking authors and sources while excluding relevant studies published in other languages. Consequently, the findings may not fully capture important research contributions from non‐English contexts. Future assessments of research on African trypanosomiasis should focus on reducing biases and overcoming these limitations. Notwithstanding, the study′s applicability and validity are unaffected, providing insightful information on the present and potential future paths of trypanosomiasis research in Africa.

## 5. Conclusion

The present analysis used bibliometric approaches to examine the general trends in African trypanosomiasis research, country performance and collaborations, affiliations, sources, and author contributions, most cited documents, and keyword evolution from 1903 to 2024. This review showed that the African trypanosomiasis research field is still emerging, with contributions from 8849 authors (affiliated with diverse institutions across the globe) across 4,036 documents. With an annual rise of 3.52%, the body of knowledge is growing gradually. Furthermore, the international coauthorship percentage (47.94%) highlights significant intercountry networking in African trypanosomiasis research. Kenya and the United Kingdom were the most productive and most cited countries, respectively. Based on the country of publication of the corresponding author, Nigeria was the most relevant country. Similarly, ABU, Zaria, Nigeria, was the institution with the highest scientific publications in the field. Although *Acta Tropica* was the most relevant source, *PLOS Neglected Tropical Diseases* had the highest impact. Buscher, P., was the most productive and the most impactful author in the field. *Antitrypanosomal* and *molecular docking* are some recent concepts that are finding relevance in the research field. For future bibliometric analyses, researchers should consider combining data from multiple databases to achieve a balanced perspective while clearly stating the rationale behind the selections. This can provide both breadth and depth in understanding research trends. This bibliometric study has unraveled the current dynamics that can direct future exploration of the field of trypanosomiasis research and has put forward suggestions for combating this neglected tropical disease as a public health threat.

## Author Contributions

**Conceptualization**: Israel Ogwuche Ogra; **methodology**: Israel Ogwuche Ogra, Emohchonne Utos Jonathan, Jeremiah Zaphnathpaaneah Adaji, Samson Usman, and Kodjovi Sossou; **formal analysis:** Israel Ogwuche Ogra; **writing—original draft preparation:** Jeremiah Zaphnathpaaneah Adaji, Benita Kenechukwu Nwajiani, and Israel Ogwuche Ogra; **writing—review and editing:** Israel Ogwuche Ogra; **supervision**: Alexander Kurovsky and Emmanuel Oluwadare Balogun.

## Funding

No funding was received for this manuscript.

## Ethics Statement

The authors have nothing to report.

## Consent

The authors have nothing to report.

## Conflicts of Interest

The authors declare no conflicts of interest.

## Data Availability

Data are available on request from the authors.
